# Addressing H-Material Interaction in Fast Diffusion Materials—A Feasibility Study on a Complex Phase Steel

**DOI:** 10.3390/ma13204677

**Published:** 2020-10-20

**Authors:** Agustina Massone, Armin Manhard, Andreas Drexler, Christian Posch, Werner Ecker, Verena Maier-Kiener, Daniel Kiener

**Affiliations:** 1 Materials Center Leoben, Forschungs GmbH, Roseggerstrasse 12, 8700 Leoben, Austria; Christian.Posch@mcl.at (C.P.); werner.ecker@mcl.at (W.E.); 2 Department Materials Science, Chair of Materials Physics, Montanuniversität Leoben, Jahnstrasse 12, 8700 Leoben, Austria; daniel.kiener@unileoben.ac.at; 3 Max-Planck-Institut für Plasmaphysik, Boltzmannstr. 2, D-85748 Garching, Germany; manharar@ipp.mpg.de; 4 Institut für Werkstoffkunde, Fügetechnik und Umformtechnik, Technische Universität Graz, Rechbauerstrasse 12, 8010 Graz, Austria; andreas.drexler@tugraz.at; 5 Department Materials Science, Chair of Physically Metallurgy and Metallic Materials, Montanuniversität Leoben, Roseggerstrasse 12/Max-Tendler-Strasse 9, 8700 Leoben, Austria; verena.maier-kiener@unileoben.ac.at

**Keywords:** advanced high-strength steels, hydrogen embrittlement, in-situ testing, scanning electron microscopy, plasma charging

## Abstract

Hydrogen embrittlement (HE) is one of the main limitations in the use of advanced high-strength steels in the automotive industry. To have a better understanding of the interaction between hydrogen (H) and a complex phase steel, an in-situ method with plasma charging was applied in order to provide continuous H supply during mechanical testing in order to avoid H outgassing. For such fast-H diffusion materials, only direct observation during in-situ charging allows for addressing H effects on materials. Different plasma charging conditions were analysed, yet there was not a pronounced effect on the mechanical properties. The H concentration was calculated while using a simple analytical model as well as a simulation approach, resulting in consistent low H values, below the critical concentration to produce embrittlement. However, the dimple size decreased in the presence of H and, with increasing charging time, the crack propagation rate increased. The rate dependence of flow properties of the material was also investigated, proving that the material has no strain rate sensitivity, which confirmed that the crack propagation rate increased due to H effects. Even though the H concentration was low in the experiments that are presented here, different technological alternatives can be implemented in order to increase the maximum solute concentration.

## 1. Introduction

One of the main goals in the automotive industry is the reduction of weight while maintaining an adequate strength and toughness, at low cost, and enhancing both safety and fuel economy. In this scenario, advanced high-strength steels (AHSS) are excellent candidates for this application, since they combine both high strength and low weight [1,2]. The most important ones are dual phase (DP), ferritic-bainitic (FB), martensitic, transformation-induced plasticity (TRIP), and complex phase (CP) steels. DP steels consist of martensitic islands in a ferritic matrix and they combine low yield strength with high ultimate tensile strength. FB steels have a microstructure of fine ferrite and bainite and strengthening is obtained by both grain refinement and second phase hardening with bainite. Martensitic steels show the highest tensile strength level and they are often subjected to post-quench tempering in order to improve ductility. TRIP steels exhibit superior strength and good formability as a result of the transformation of metastable retained austenite to martensite during deformation. The microstructure of CP steels contains small amounts of martensite, retained austenite, and pearlite within a ferrite/bainite matrix. An extreme grain refinement is created in this material by retarded recrystallization or precipitation of microalloying elements, such as Ti or Nb [3].

Despite their remarkably good mechanical properties, AHSS are susceptible to hydrogen embrittlement (HE), and this can lead to a loss of ductility. When AHSS are electroplated with a sacrificial metal, typically Zn, H can be absorbed during the coating deposition, as the process is not 100% efficient. Moreover, if the sacrificial coating corrodes in service, the exposed areas of the steel substrate will act as cathodic sites and H can be absorbed into the material [4]. H can also be introduced into the material during the painting process of a body in white structure. Lovicu et al. [5] measured the H content absorbed during the production process of autobody components, in which cathodic reactions in water solution take place in the phosphatizing and electrophoresis stages of the painting process. During these reactions, atomic H can form on the steel surface and diffuse into the material. The absorbed H during the painting process was lower than about 0.4 wppm. In this context, H is one of the main limitations in the use of AHSS, since it can reduce the ultimate tensile strength, ductility, fatigue strength, and/or fracture toughness of the steels [6]. This degradation becomes apparent when the material is under residual or applied tensile stresses. Absorbed H diffuses through the metal facilitating crack propagation and, the higher the stress level of the material, the more susceptible it is to undergo detrimental HE effects [7].

The mechanism of HE has been under discussion for many years, leading to different interpretations and controversial findings [8,9,10,11,12,13,14,15,16]. Nevertheless, it is accepted that HE can only occur for a critical combination of local stress and local H concentration [6,17,18,19]. This critical value for H concentration may depend on applied stress, microstructure, trapping state, and tensile strength level, among others. Moreover, it is believed that there is a saturation level above which there is a minimal change in HE susceptibility [17]. Drexler et al. confirmed that this is the case for AHSS [18,19].

High-strength materials are more prone to HE due to an increased number of potential fracture initiation sites, with martensite usually being the most susceptible steel phase [7]. The morphology of H-assisted fracture in AHSS depends on the steel microstructure and H concentration and it can be either unaffected or changed from ductile microvoid coalescence to quasi-cleavage, cleavage, or intergranular failure [17,18,19,20].

The effect of H on high-strength steels has been widely studied [5,6,18,19,20,21,22,23,24,25,26,27,28,29,30,31,32,33,34,35], but there are few studies regarding the interaction between H and CP steels. Malitckii et al. [32] investigated the role of retained austenite in CP steel and proposed that fatigue intergranular areas might be formed due to H accumulation at the austenite/martensite interfaces, followed by H-induced decohesion. Loidl et al. studied the effect of H on a CP1200, among other different AHSS. They showed that the degree of embrittlement was similar to martensitic steels and that TRIP steels presented the highest tendency to HE [33]. Lovicu et al. [5] concluded that martensitic microstructures exhibit great susceptibility to HE and, the higher the tensile strength, the lower the critical H concentration to produce damage in the material. Duprez et al. [29] studied the effect of H on a DP steel, a TRIP steel, a FB steel, and a ferrite-cementite grade. They demonstrated that the ductility of all the samples was reduced after electrochemical charging, with TRIP and DP steels the most susceptible ones. Nevertheless, after discharging the samples for one week, a large part of the ductility was recovered. This proved that the damage was caused by the intrinsic presence of H and not by an irreversible damage mechanism. Rehrl et al. [34] investigated the effect of different loading rates in four grades of AHSS and showed that, at high strain rates, there was no effect of H on the mechanical properties. Only with slow strain rate testing, the elongation at failure was reduced. This was explained when considering that at a high strain rate, the H diffusion is too slow to reach highly stressed microstructure regions. Drexler et al. studied the local H accumulation and its effect on HE for cold formed, punched, and heat treated CP1200 and DP1200 [18,19,20].

In all of the studies mentioned above, the investigations were made either with ex-situ H charging or without in-situ observation. These two approaches can lead to a misinterpretation of the results. When the materials are ex-situ charged, there is a risk of H outgassing before the test is performed, especially in some steels where H diffusion is very fast [35]. Without in-situ observation, the H effect can only be analysed post-mortem and not during the test. Even though the effect on mechanical properties can be determined, details on crack dynamics cannot be investigated without an in-situ approach. In order to overcome these limitations, several studies have already been made with in-situ charging [36,37,38,39,40] and, in the present work, the interaction between H and a CP steel was investigated by implementing an in-situ method, which allows for in-situ H charging during a tensile test inside a scanning electron microscope (SEM).

## 2. Materials and Methods 

### 2.1. Material

A CP1200 steel was investigated in this work. The main microstructural phases are martensite, tempered martensite, and bainite, with a small content (<2%) of retained austenite. Table 1 shows the chemical composition of the material.

**Figure 1:**
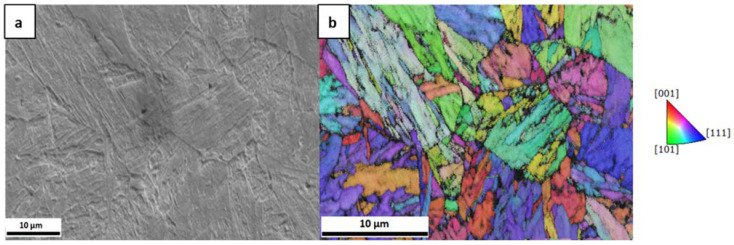
(**a**) Secondary electron image; and (**b**) inverse pole figure in the normal direction of the investigated CP1200 microstructure.

The microstructure analysis was conducted on a field emission SEM Zeiss LEO 1525 (Carl Zeiss GmbH, Oberkochen, Germany) while using an acceleration voltage of 20 kV and by EBSD using a pixel size of 59 nm and a working distance of 15.7 mm. Figure 1 depicts the microstructure of the material. Figure 1a exhibits the secondary electron image and Figure 1b shows the inverse pole figure map in the normal direction of the same region. The average prior austenite grain size was measured to be ~3 µm.

The as-delivered material had a thickness of 1.2 mm and tensile samples with the geometry of 32 mm length, 5 mm width, and 0.5–1.10 mm thickness were fabricated by electrical discharge machining parallel to the rolling direction. The samples were then ground and polished with 9 μm, 3 μm and 1 μm diamond paste to remove surface damage from machining. In the centre of the sample, in a rounded area of 4 mm diameter, the thickness was reduced to ~120–200 µm to reach steady-state permeation through the thickness of the charged samples more quickly. Table 2 displays the thicknesses of the samples.

In-situ mechanical tests were performed in an SEM Zeiss Stereoscan 440 (Carl Zeiss GmbH, Oberkochen, Germany) operating at an acceleration voltage of 10 kV for imaging. A Kammrath & Weiss tensile stage (Kammrath & Weiss GmbH, Dortmund, Germany) equipped with a 10 kN load cell and an inductive displacement sensor was used for the tensile tests. The accuracy of the load cell is in the order of ~1 N, and the accuracy of the displacement sensor is ~0.5 μm.

The effect of strain rate was studied by tensile testing uncharged specimens with nominal strain rates of 3 × 10^−5^ s^−1^, 1.5 × 10^−4^ s^−1^, and 3 × 10^−4^ s^−1^. For these tests, samples with uniform thickness and no thinned area were used. To further study the strain rate sensitivity and measure depth-dependent properties, nanoindentation testing was performed on a KLA G200 platform nanoindenter (KLA, Milpitas, CA, USA) that was equipped with a three-sided diamond Berkovich tip (Synton-MDP). Six constant strain rate indentations with an applied indentation strain rate of 0.05 s^−1^ and additionally five strain-rate jump tests with a strain-rate profile of 0.05 s^−1^, 0.005 s^−1^, 0.05 s^−1^, 0.001 s^−1^, and 0.05 s^−1^ (applied changes in the strain rate every 500 nm) were executed [41]. The continuous stiffness measurement technique was utilized to continuously measure the contact stiffness and, thereby, the hardness and Young’s modulus over indentation depth. This technique involves applying a dynamic load that is then used to measure the stiffness, which is further processed in order to calculate the modulus and hardness of the material. This method allows for the measurement of the depth-dependent properties of materials [41].

### 2.2. Plasma H Charging

The effect of H-material interaction was studied on the CP steel while using a dedicated in-situ design. H was charged into the material via localised loading by a deuterium plasma cell, allowing for the combination of in-situ charging, mechanical testing, and observation in an SEM. The applied method consists of a miniaturized radio frequency-plasma cell, in which two electrodes are confined in a vacuum vessel and deuterium gas is supplied. The tensile sample, operating as the grounded electrode, can, in this way, be charged from the bottom side with the H isotope, providing a contamination free top surface for observation. Only the thinned centre of the samples was charged and the observation was localized in this region. The main advantage of this method, when compared to conventional electrochemical charging, is that it allows having a high-resolution observation during H charging and deformation. Furthermore, there is no risk for the SEM, since the plasma turns off automatically when the sample fractures. For more details of the method, see ref. [40].

The influence of different H charging conditions was examined by applying different power settings, obtaining plasmas with different bias voltages. A higher bias voltage can be correlated with a higher ion flux and implantation range and, consequently, higher ion concentrations. Power levels of 5 W, 6 W, 8 W, and 11 W were applied, resulting in DC bias voltages of 110 V, 127 V, 173 V, and 174 V, respectively. All of the samples were consistently only charged during the tensile test, i.e., no pre-charging was performed. It was demonstrated [42] that, in the presence of H, the reduction of fracture area increases with decreasing deformation rate and that only with slow strain rate testing the elongation to failure of a material can be decreased [34]. With a slow deformation rate, diffusible H has more time to migrate towards the crack tip, which results in an embrittlement of the material. For this reason, the tensile tests were performed with a slower strain rate than the previous cases, using a displacement rate of 0.1 μm/s. Afterwards, the fracture surfaces were observed in the FEG-SEM LEO 1525.

A sample was charged for 4 h and then discharged for 12 h before starting the test in order to address the potential reversibility of HE and the possibility of plasma damage. The results were subsequently compared with an uncharged specimen.

Moreover, maintaining plasma parameters fixed, the effect of pre-charging time was investigated. Three samples were tested: an uncharged sample for reference and two charged samples with 3 h and 6 h of H pre-charge, respectively. After the pre-charging time, the charging was also maintained during the tensile test (~1 h duration). Afterwards, the surfaces were analysed using EBSD and fractography was performed with the FEG-SEM using the previously mentioned parameters. 

### 2.3. H Uptake and Diffusion Simulation

The model described in [43] was utilised in order to assess the present H concentration. This is a model for ion-driven permeation of H in plasma-facing materials at steady state. In steady-state, the incident flux is balanced with the reflected and permeated fluxes. Depending on the relative rate of recombination and diffusion on front and back sides of the membrane, there are three possible ion-driven permeation processes: diffusion-limited on both sides (DD regime), recombination-limited on both sides (RR regime), and recombination-limited on one side and diffusion-limited on the other side (RD regime). Equation (1) shows the estimation of the maximum concentration for a RR and RD regimes. In these regimes, the surface recombination is the rate-limiting step and is valid for fast-diffusion materials. Because the parameters on the back side do not affect the maximum concentration at steady state [43], the equation is the same for both cases.

(1) $$C_{max}=\surd \frac{\eta }{k_{f}}$$

where *C_max_* is the maximum lattice H concentration, *η* the ion flux, and k_f_ the recombination coefficient. The ion flux was calculated from Langmuir Probe measurements and it is described in detail in [40]. A Monte-Carlo program, SDTrim.SP 6.0 [44] was used for estimating the implanted fraction/particle reflection yield and the mean implantation range. The value of k_f_ was calculated according to three different sources [43,45,46].

For a more detailed analysis, the H concentration was calculated using a more sophisticated diffusion model [47,48], developed and implemented as subroutine (UMATHT) in the finite element simulation software package Abaqus (version 2019) [49]. Newton–Raphson scheme is used to solve the system of equations, whereas Crank–Nicholson procedure is used for the time integration. This is a sequentially coupled diffusion-mechanical model, which considers concentration driven diffusion, stress driven diffusion, as well as physically meaningful boundary conditions. The interplay between trapped and lattice H is considered by the following relationship written in its multiple trap formulation [50]:

(2) $$\frac{\gamma_{lattice}\left(1-\gamma_{trap,k}\right)}{\gamma_{trap,k}\left(1-\gamma_{lattice}\right)}=\exp\left(-\frac{\Delta E_{k}}{R_{g}T}\right),\;\;\;\;\;\left(k=1,\ldots ,m\right)$$

**Figure 2:**
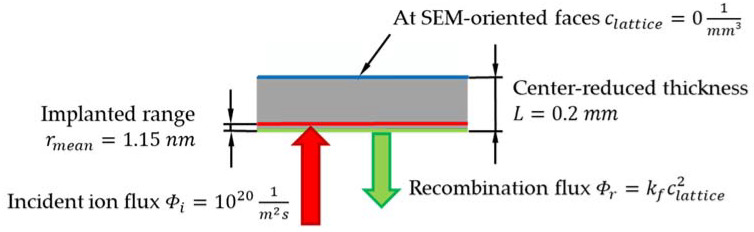
RD boundary conditions of the one-dimensional (1D) hydrogen permeation model. The time history of the sum of the incident ion flux and the recycling flux is applied as boundary condition at the H charging area in the three-dimensional (3D) model.

With $\gamma_{lattice}$ as site fraction of lattice H, $\gamma_{trap,k}$ as trap site fraction and $\Delta E_{k}$ as trapping energy for the k^th^ sort of trap. The parameters of the model can be either determined from permeation experiments [51,52] or from thermal desorption spectroscopy measurements [53,54]. The applied model for the CP1200 steel only considers one effective trap with a trap energy of 30 kJ/mol and a trap density of 5.7 × 10^−7^ [18]. A trapping energy of about 30 kJ/mol is representative for dislocations and some kinds of grain boundaries and it is related to rather shallow traps. The three-dimensional (3D) sample geometry was modelled making use of a quarter symmetry and it was discretised by finite elements with linear shape functions (DC3D8) and an element size of interest of 2 × 10^−3^−2 × 10^−2^ mm, resulting in a number of 13 elements alongside the flux direction. A mesh convergence study was done in order to avoid mesh dependency of the results. The applied flux boundary condition on the plasma-oriented surface in the 3D model results from the incident ion intake flux $\phi_{i}$ of 10^20^ m^−2^s^−1^ in 1.15 nm depth and on the recombination flux $\phi_{r}=k_{f}c_{lattice}^{2}$, with $c_{lattice}$ being the locally corresponding lattice hydrogen concentration. Due to narrow distance between the flux due to plasma loading and the recombination flux, the one-dimensional (1D) hydrogen permeation FE model calculated net influx is used to prescribe the hydrogen intake in the 3D model. Figure 2 depicts the boundary conditions applied to the 1D hydrogen permeation model. In fact, the boundary conditions on the SEM-oriented face of the sample was chosen corresponding to, both, the RR ($flux=k_{f}c_{lattice}^{2}$) and the RD ($c_{lattice}=0$) regimes, and the differences were negligible. Therefore, only the model and results of the RD case are shown in the present paper.

## 3. Results

### 3.1. Rate Dependance of Flow Properties

**Figure 3:**
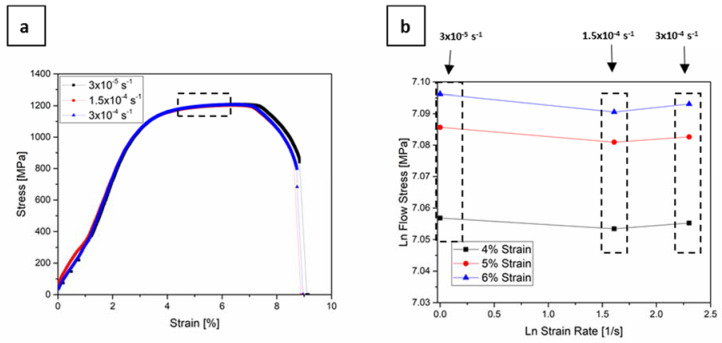
(**a**) Stress-strain curves of CP 1200 steel tested strain rates of 3 × 10^−5^ s^−1^, 1.5 × 10^−4^ s^−1^, and 3 × 10^−4^ s^−1^; (**b**) ln flow stress-ln strain rate plot for the strains indicated in the box in (**a**), showing no significant strain rate sensitivity.

Figure 3a shows the stress–strain curves of three specimens that were tested with 3 × 10^−5^ s^−1^, 1.5 × 10^−4^ s^−1^, and 3 × 10^−4^ s^−1^. The initial part of the curve that corresponds to 3 × 10^−5^ s^−1^ strain rate does not start with zero stress, due to possible friction effects, but after approximately 1% strain, the curve exhibits a normal behaviour. There is almost no difference between the three curves; the yield stress is ~1060 MPa, the tensile strength ~1200 MPa, and strain to failure ~9%. The strain rate sensitivity of a material can be verified by the value of the strain rate sensitivity index, m, from a simple power-law relationship [55]:

(3) $$\sigma =\varepsilon^{m}$$

where σ is the flow stress and ε the strain rate. The value of m can be then determined as the slope of the plot of lnσ vs. lnε. Figure 3b depicts the flow stress vs. strain rate for the strains that are indicated in the box in Figure 3a. The calculated *m* for three curves was almost zero, proving that the material exhibits no significant strain rate sensitivity in the investigated strain rate regime. 

**Figure 4:**
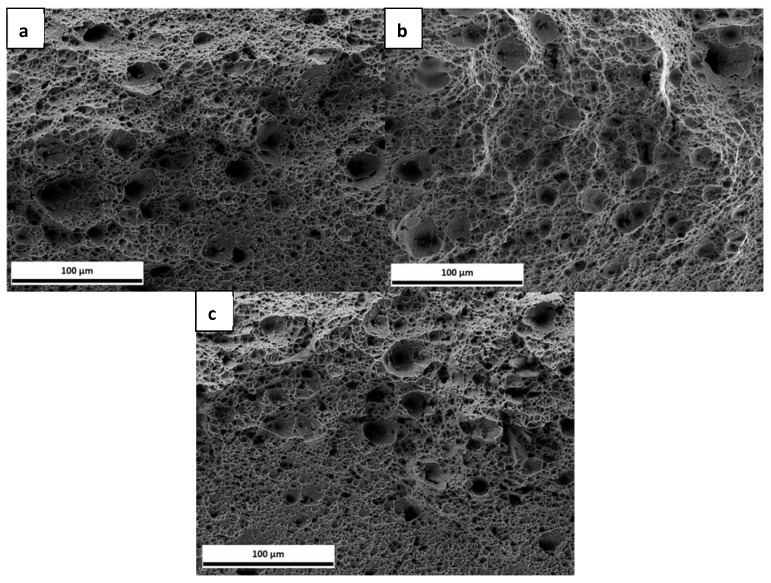
Fracture surfaces of complex phase (CP) steel tested with strain rates of (**a**) 3 × 10^−5^ s^−1^; (**b**) 1.5 × 10^−4^ s^−1^; (**c**) 3 × 10^−4^ s^−1.^

At a macroscopic level, the three samples exhibited a high degree of ductility (necking). Figure 4 shows the fracture surfaces of the three specimens, where the same ductile failure behaviour was observed for all specimens, i.e., microvoid coalescence, leading to the presence of dimples on the fracture surfaces. The size of approximately 50 dimples in each of the three samples were estimated from the SEM images, giving a bimodal distribution with sizes of 14.5 ± 0.8 µm and 6.6 ± 0.6 µm. No effect of strain rate was observed in the tested range. 

**Figure 5:**
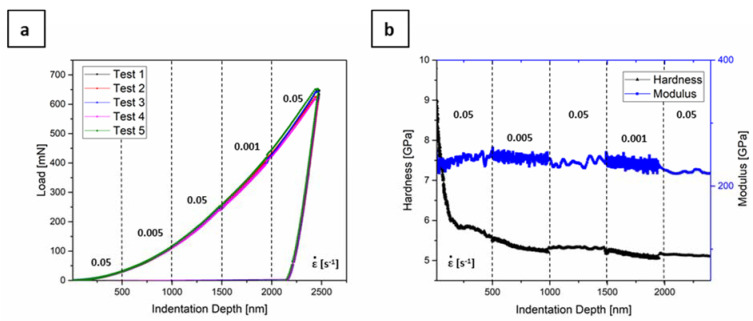
(**a**) Load-indentation depth plots corresponding to jump tests with 0.05 s^−1^, 0.005 s^−1^, 0.05 s^−1^, 0.001 s^−1^, and 0.05 s^−1^, with a change in the strain rate every 500 nm; (**b**) the exemplarily resulting hardness and Young’s modulus.

From the constant strain rate indentation tests, the average hardness and Young’s Modulus were calculated to be 5.13 GPa and 233 GPa, respectively. Similar hardness values were reported for tempered martensite and martensitic steels with similar C content [56,57]. Figure 5a depicts the load-indentation depth plot that corresponds to the strain rate jump tests and Figure 5b shows the exemplarily resulting hardness and Young’s modulus over indentation depth. The Young’s modulus is independent of the applied strain rate and the differences in data points is due to the differences in testing times with different strain rates. The hardness levels clearly shift with strain rate, even though by only a small amount. The decreasing hardness at very shallow indentation depths is related to the indentation size effect [58], but it does not affect the analysis of strain rate sensitivity. The calculation of the strain rate sensitivity m in nanoindentation experiments is made with the hardness, which is directly related with the stress through Equation (4):

(4) $$\sigma =H/C^{*}$$

where *σ* is the flow stress, *H* the hardness and C^*^ a constraint factor. The calculated strain rate sensitivity *m* was on average 0.006 ± 0.0005. This result confirms that the material exhibit almost no strain rate sensitivity within the tested parameter range.

### 3.2. Effect of H Charging

#### 3.2.1. Effect of Pre-Charging Time

**Figure 6:**
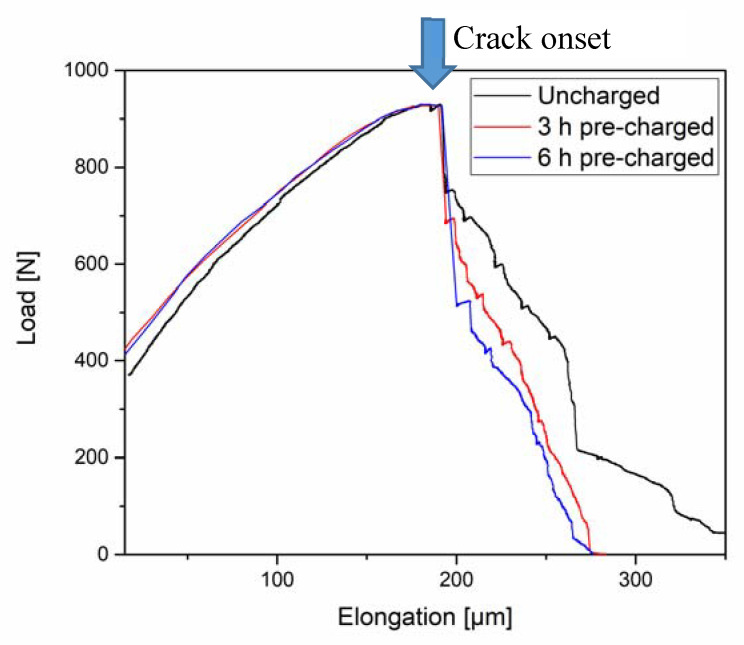
Load-elongation curves of three CP steel samples: uncharged, 3 h, and 6 h pre-charged.

Figure 6 depicts the load-elongation curves of an uncharged sample and two charged samples with different pre-charging times, both also continuously charged during the tensile test. The first linear part of the curve is not affected by the presence of H. However, a trend can be observed in the plastic part, after the crack onset, which is indicated by an arrow. With increasing H-charging time, the elongation to failure decreased slightly, and there was a more pronounced drop in the load at ~ 200 μm, which is when cracking was initiated. 

**Figure 7:**
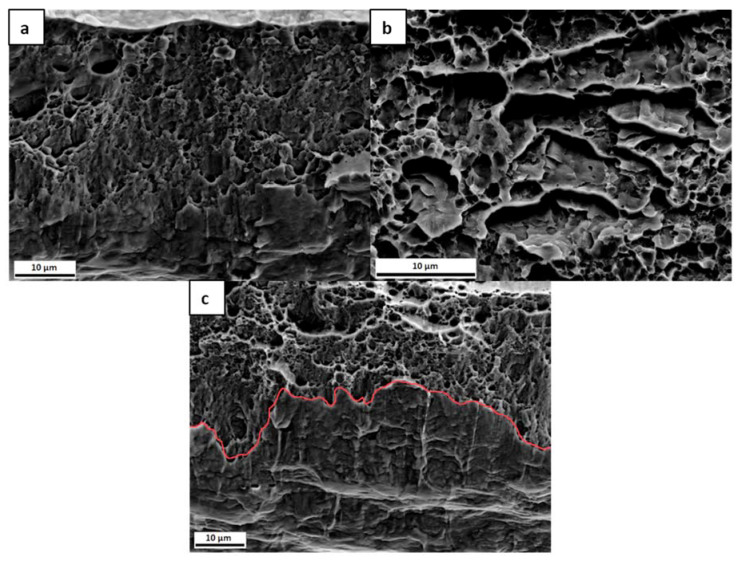
Fracture surfaces of the (**a**) uncharged; (**b**) 3 h pre-charged; and, (**c**) 6 h pre-charged CP steel samples.

In Figure 7, the fracture surfaces of the three samples are presented. The uncharged sample, Figure 7a, exhibits a ductile failure. When comparing with the uncharged samples from Figure 4, they do not look very similar, since, in this set of samples, the thickness was reduced in the centre of the sample, as described above, which gives rise to a somewhat different appearance of the fracture surface. The charged samples presented in Figure 7b,c, on the other hand, exhibit some differences when compared to the uncharged specimen. In Figure 7b, in addition to voids, some flat regions can be seen. In the sample with the longer charging time, Figure 7c, there is a marked difference between the upper and lower part of the fracture surface, as indicated with a red line. Only in the upper part of the surface microvoids can be observed, while the lower part, where the H was supplied, shows another failure mechanism with more localized damage. Even though the lower part of Figure 7a also exhibits some surface damage, this is less pronounced than for the charged sample.

**Figure 8:**
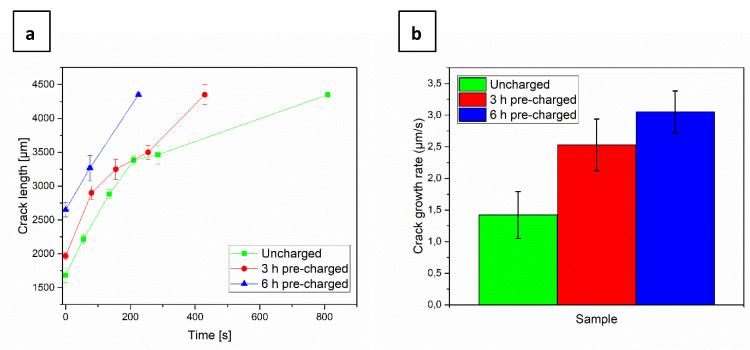
(**a**) Normalized crack length evolution; (**b**) crack growth rate of the uncharged, 3 h pre-charged and 6 h pre-charged CP steel samples.

From in-situ SEM images that were recorded during the tests, the linear intercept of the crack length was estimated at different loading steps for each sample, as shown in Figure 8a. For comparison purposes, the crack initiation times were normalized to 0 s. When comparing the slopes of the curves for each sample, it can be seen that the crack propagation rate increased with charging time. Figure 8b shows a linear estimate of the crack growth rate for the three samples. While the crack growth rate for the uncharged sample was ~1.5 µm/s, the 3 h pre-charged sample exhibited a rate of 2.5 µm/s, while, for the 6 h pre-charged one, it further increased to 3 µm/s. 

**Figure 9:**
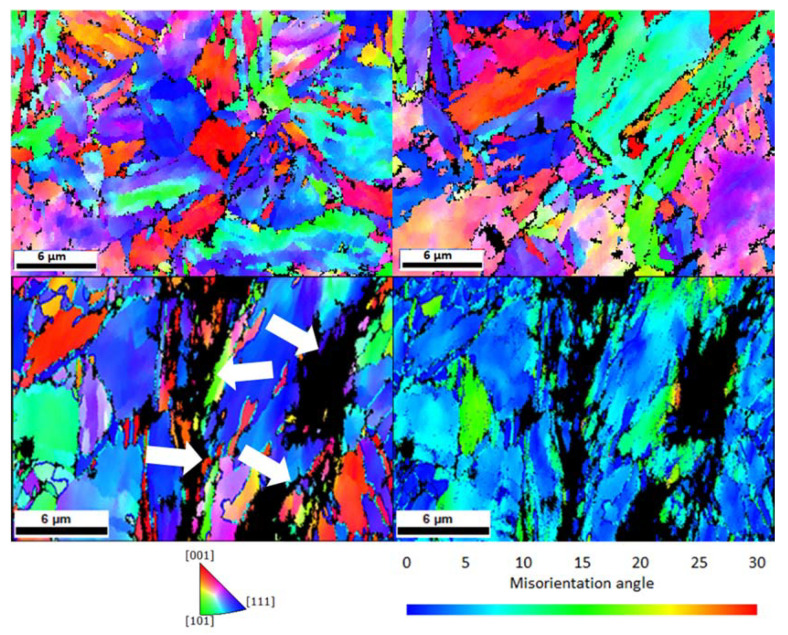
Inverse Pole Figure maps in the normal direction of the (**a**) uncharged, (**b**) 3 h pre-charged, (**c**) 6 h pre-charged CP steel samples near the fracture and (**d**) misorientation map of 6 h pre-charged CP steel sample.

The surface of samples in regions near the fracture site was analysed using EBSD. The Inverse Pole Figure maps in the normal direction are shown in Figure 9. Secondary cracks, highlighted with arrows, can only be seen in the sample with the longer charging time, Figure 9c,d. Nevertheless, while the secondary cracks seem to follow interfaces in Figure 9c, it was not possible to determine whether the cracks started at grain boundaries or inside the grains. No secondary cracks were observed in the uncharged and 3 h pre-charged samples. 

#### 3.2.2. Effect of Plasma Parameters

**Figure 10:**
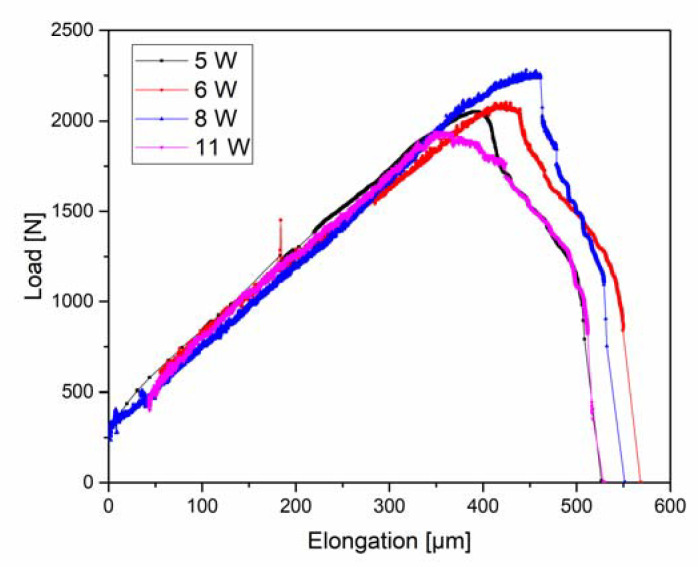
Load-elongation curves of CP 1200 steel tested under different plasma charging conditions. Power levels of 5 W, 6 W, 8 W, and 11 W were applied for running the plasma.

Figure 10 shows the load-elongation curves of samples tested under different plasma conditions. The noise that was observed in some of the curves was due to interference between the radio frequency power supply and the electronics in the tensile module, despite the shielding of the cables. While inconvenient, this does not affect the data. It is important to mention that, since the samples used for each case study correspond to a different machining set, the difference in thickness gives rise to different maximum loads. 

The linear loading regimes of the curves are approximately the same for all tested samples. Nevertheless, there are some differences in the maximum load (tensile strength) and elongation to failure. It was expected that the samples with higher power and, therefore, higher H concentration, would be the first ones to fail, but no clear trend was observed. Because load is plotted instead of stress due to the locally thinned sample geometry, the differences in tensile strength could be attributed to small differences in the thickness of the samples due to fabrication. Even a slight difference of ~0.02 mm in the thicknesses of the thinned area of the samples tested could lead to an error of around 5.5% and 3.5% in the maximum load and elongation to failure, respectively, which is consistent with the differences observed in Figure 10. Considering that the H concentration increases with the power (a higher power results in a higher plasma bias voltage), the sample that was tested with 11 W should have the smallest elongation to failure, while the sample with 5 W, the largest. In Figure 10 it can be seen that, even though the sample with 11 W has lower elongation to failure than the samples with 6 W and 8 W, the one with 5 W has approximately the same. 

**Figure 11:**
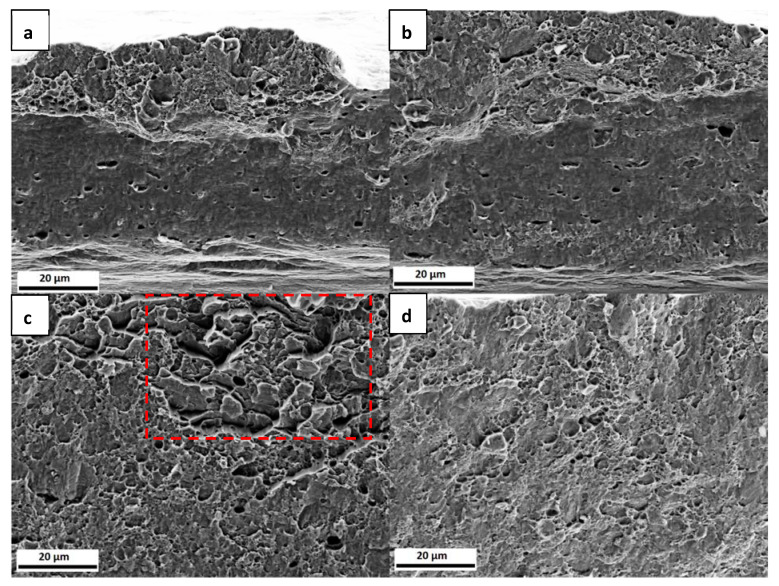
Fracture surfaces of the CP steel specimens tested with (**a**) 5 W; (**b**) 6 W; (**c**) 8 W; and, (**d**) 11 W. The red box in (**c**) indicates an area with shear fracture or specially oriented grains.

Figure 11 displays the fracture surfaces of the tested samples within the reduced thickness area. Although all of them failed in a ductile manner, they exhibited less degree of necking than the uncharged specimens and there were some differences at the microscopic level. Figure 11a,b shows a very similar morphology. While the upper part of the surfaces exhibit the presence of dimples, the lower parts show a rather smooth surface with less dimples. Figure 11c, on the other hand, shows a mixture of dimples with areas (marked with a box) that could be shear fracture or grains specially oriented. In Figure 11d, the fracture surface morphology is more uniform than the previous cases, being dominated by the presence of small dimples. 

#### 3.2.3. Effect of H Charging-Discharging

**Figure 12:**
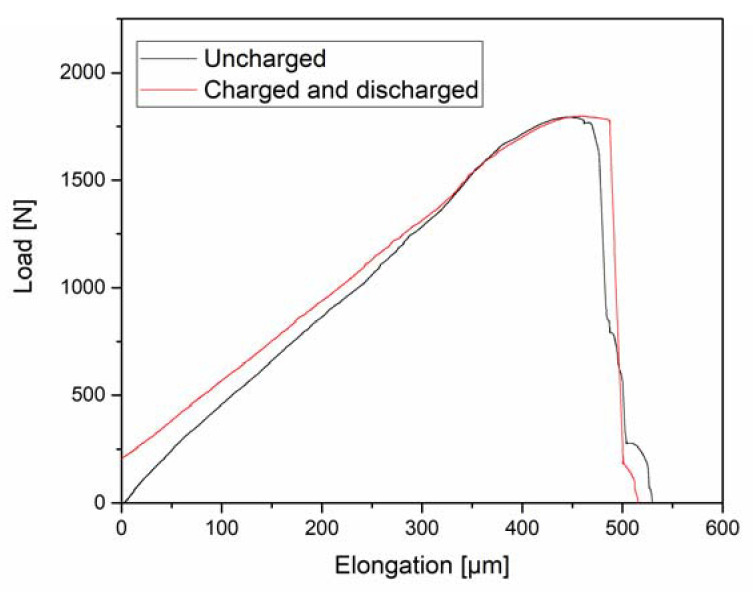
Load-elongation curves of an uncharged CP 1200 steel sample and one initially charged for 4 h and tested after discharging for additional 12 h in vacuum.

In Figure 12, the stress-strain characteristic of an uncharged sample is compared to a sample that was charged and tested after a discharging time of 24 h. There are some differences in the data, but these are related to small dimensional differences. This would support the notion that HE is a reversible effect. Fractography was also performed to fully support this statement. 

**Figure 13:**
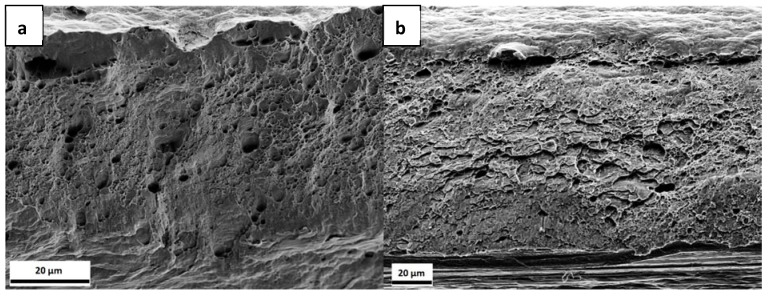
Fracture surfaces of the (**a**) uncharged and (**b**) charged and discharged CP steel specimens.

Figure 13 shows fracture surfaces corresponding to the uncharged and charged and discharged samples. It can be seen that, while the uncharged sample exhibits a typical ductile microvoid coalescence failure, see Figure 4, the charged and discharged sample display some regions where the morphology is similar to the one presented in Figure 11c, with a mixture of dimples and more flat features.

### 3.3. H Concentration

The Monte-Carlo program STrim.SP 6.0D gave a r_mean_=1.15 nm and that 41% of the particles are implanted. From Langmuir Probe measurements [40], the ion flux was calculated to be 10^20^ m^−2^s^−1^. Table 3 shows the recombination coefficients that were calculated according to refs. [43,45,46] and the resulting H concentration. Because the different sources resulted in different concentration values, it is considered that they give a range for H concentration. It is important to mention that these values correspond to lattice H, i.e., the analytical calculation does not consider the trapping effect and, therefore, a higher concentration is expected in the material.

**Figure 14:**
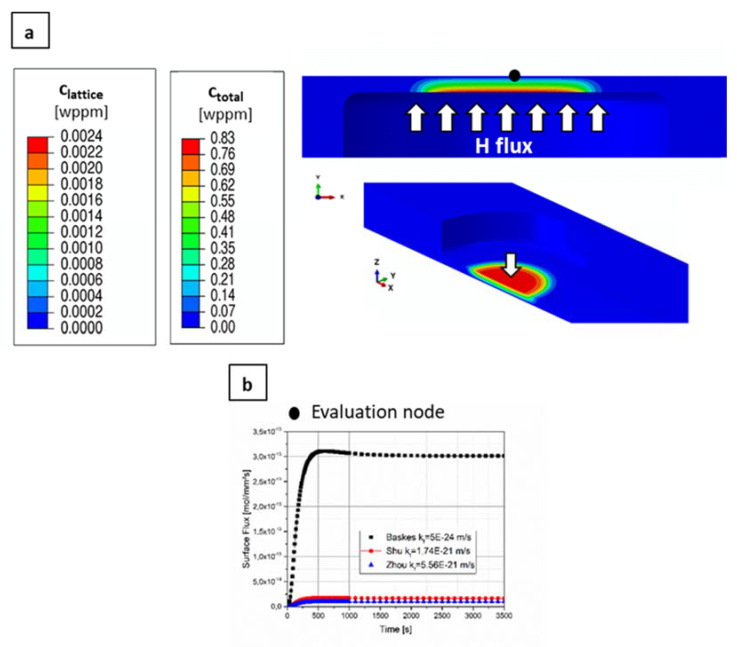
(**a**) H concentration distribution through the thickness of the CP steel sample, the arrows indicate the direction of H charging from the bottom of the samples; and, (**b**) H flux over time in an evaluation node on the top surface of the sample.

Figure 14 shows more elaborated simulation results. Here, Figure 14a depicts the cross section with two different views of the tensile samples, where the arrows indicate the direction of H charging. The concentration decreases through the thickness of the sample, resulting in 0 wppm at the top surface. The lattice concentration shows very good agreement with the analytical calculation made with ref. [46]. However, the total H concentration summing over the H stored in the interstitial lattice positions and in H traps reaches 0.82 wppm, and it is around a factor of 350 higher than the corresponding lattice H. Figure 14b shows the corresponding flux for the three sources in the evaluation node. Even though the values differ, the three cases show that a steady state is reached after approximately 500 s.

## 4. Discussion

The H degradation behaviour is known to be dependent on the concentration of the absorbed H [58]. Zackroczmski et al. studied the effect of H concentration on a duplex stainless steel and concluded that the intensity of HE was strongly dependent on the concentration of the absorbed H [59]. Furthermore, HE does not occur below a critical H concentration value [6,17]. Generally speaking, a higher DC self-bias voltage leads to a higher ion concentration [60]. This means that, increasing the power supplied to the RF discharge, an increase in the H concentration should be obtained. Taking this into account, it was expected that, with increasing power in Figure 10, the total elongation (related to the ductility of the specimens) should decrease. As mentioned before, this behaviour was not observed in the samples that were tested with 5 W, 6 W, 8 W, and 11 W, and considering that the accuracy of the displacement sensor is ~0.5 µm, the small differences in elongation to failure could be attributed to the 3.5% error arising from the slight differences in thickness.

Although there were no marked differences in the load-elongation curves, the fracture surfaces exhibited different characteristics. When comparing with the fracture surfaces of the uncharged samples shown in Figure 4, there are some differences with the charged samples in Figure 11. The uncharged samples exhibit a typical ductile failure, formed by the coalescence of large voids involving the nucleation and growth of small voids leading to the presence of large and small dimples on the fracture surface with a bimodal distribution [61]. On the other hand, although, in the charged samples, dimples can also be observed, these have smaller size than in the uncharged case. The dimple size of the samples tested with 5 W and 6 W, as estimated from the SEM images, was 1.4 ± 0.2 µm. The sample tested with 8 W exhibited a dimple size of 2.4 ± 0.5 µm and the 11 W sample, 1.8 ± 0.4 µm. While these values do not follow a specific trend, they are smaller than the uncharged specimens, which presented a bimodal distribution with sizes of 14.5 ± 0.8 µm and 6.6 ± 0.6 µm, as before mentioned. This is in agreement with many previous studies, which showed that the presence of H reduces the dimple size on the fracture surface [58,59,60,61,62,63,64,65,66].

Moreover, the samples depicted in Figure 11a,b show similar features: the lower part of the fracture surface is rather flat with less dimple features, while the upper part looks more similar to ductile failure. This flat part could be the result of the plastic deformation of the sample. Nevertheless, the dimples in this case are uniform in size and there is not a bimodal distribution present as compared to the uncharged specimens. The similarities between these two samples, with 5 W and 6 W, could be attributed to similar H concentration values. When considering that the H charging is conducted from the bottom of the sample, it is reasonable that with a gradient in H concentration, a gradient in the fracture surface morphology is obtained. In contrast to these two specimens, the ones that were tested with 8 W and 11 W exhibit a more uniform fracture surface, less plastically deformed, and also different to the ones seen in the uncharged specimens. Both of the samples have smaller dimples than the uncharged ones and the main difference between the two charged samples is that in the 8 W condition there are some with flat features. It has been reported [67] that a reduction in dimple size can be a consequence of H effect and it represents a large increase in dimple nucleation.

The load-elongation curves presented in Figure 12 demonstrate that there is either no effect on the mechanical properties caused by the presence of H, or that this effect is reversible, and the properties of the material are restored once the H is eliminated from the samples. These facts would be in line with the premise that diffusible H, which can allegedly diffuse out of the specimens at room temperature, is responsible for HE [68], while trapped H has little to no effect. It has been reported [16] that, in this material, H is mainly trapped at dislocations and martensitic lath boundaries, which are not deep traps. Therefore, it is believed that all of the H effuses when discharging the material. Anyways, the observation of the fracture surfaces in Figure 13 demonstrates that the failure characteristic is somewhat affected.

In Figure 6, the load-elongation curves depict a different trend than in the previous cases. Notably, the three specimens tested converged to approximately the same maximum load. This is mostly set by the unaffected uncharged specimen parts. However, once the failure in the thinned charged parts of the samples started, there was a larger drop in the load with increasing charging time. Furthermore, the crack propagation rate, as shown in Figure 8, also increased in the presence of H and the ultimate strain was reduced by the presence of H. Even though the crack propagation rate was accelerated with increasing charging time, the final elongation was approximately the same for the charged samples. This is in agreement with the result presented in Figure 14b, where it is shown that a steady-state is reached after a very short charging time and the H concentration does not further increase. It is also worth mentioning that the material exhibits diminishing strain rate sensitivity (see Section 3.1.), which means that the changes in crack propagation rate does not relate to different loading situations, but due to H effects on crack propagation.

The fracture surfaces in Figure 7 depict that, in the presence of H, the fracture morphology changed. In the 3 h pre-charged sample, as in Figure 7b, there are “flat” regions, while, in the sample with 6 h of H pre-charge, there is damage in the bottom part of the surface. Although none of the samples exhibited brittle fracture characteristics, the fracture morphology was affected by the presence of H for both pre-charging times, (Figure 7b,c). It is known that the morphologies of H-enhanced fracture surfaces can be changed from microvoid coalescence to quasi-cleavage, cleavage or intergranular fracture, or they can also be unaffected [69]. Furthermore, dimple size was also calculated from the SEM images for these samples, again confirming again that, in the presence of H, the dimple size was reduced. The uncharged specimen exhibited a dimple size of 3.2 ± 0.9 μm, while the sizes of the 3 h pre-charged and 6 h pre-charged samples were 1.3 ± 0.1 and 1.4 ± 0.2 μm, respectively.

To gain a better understanding on the H effect on these samples, the surfaces of the samples were also investigated through EBSD. Analysing the EBSD scans next to the fracture site, it was evident that in the 6 h pre-charged sample secondary cracks formed, and a larger amount of local misorientation in their surroundings was present, as shown in Figure 9d. Misorientation maps can be used as an approach for visualizing plastic deformation. They are a measure of the geometric dislocations in the crystalline lattice and, therefore, measures of plastic deformation at the microstructural level [70]. Although there is a lack of quantitative measures of deformation (strain, strain gradient, dislocation density, etc.), it can be locally related to the density of geometrically necessary dislocations. This indicates that in the sample with 6 h pre-charging, there is a localized plastic deformation in the areas next to the secondary cracks, which means that H could promote a greater dislocation activity in some regions of the microstructure that eventually lead to the formation of many secondary cracks.

The calculation of the present H concentration using two different approaches, a simple analytical method and a more sophisticated simulation approach, generally results in a low value. As expected, there is a gradient through the thickness of the sample, giving the lowest value at the top surface subjected to observation. On the plus side, a steady state concentration profile is reached after only 500 s. This is almost immediately after starting the charging, meaning that varying the pre-charging times would not have any effect in the results, as confirmed by the final elongation of the charged samples presented in Figure 6. Nevertheless, this does not explain why the crack propagation rate increased with charging time and why secondary cracks were only observed in the 6 h pre-charged sample. A possible explanation is that with pre-charging deeper traps are filled up causing more damage.

The low H concentrations could explain why there were no major differences in the load-elongation curves presented in Figure 10, i.e., the mechanical properties were not strongly affected. According to the simulation results that are shown in Figure 14, the maximum H concentration in the plasma-exposed surface is 0.83 wppm and Drexler et al. [18] found that for the same CP1200 steel there is a minor effect on the fracture strain at this H exposure. When considering that during body in white painting process, steel absorbs around 0.4 wppm of H content [5], it would be safe to assume that the CP steel will maintain its mechanical properties during this production process. Drexler et al. [18] showed that a pronounced HE starts at a H concentration of around 2 wppm. Nevertheless, as mentioned previously, some differences in the fracture surfaces were observed, especially when compared with the uncharged specimens, already indicating an onset on material behaviour modification at quite low H concentration, but without losing their strength and ductility.

Low H concentrations will always be a challenge for this high-diffusivity material, but different technological alternatives can be implemented in order to solve this issue. In the future, for example, impermeable nm thick coatings, transparent to the electron beam, could be used to limit the outgassing on the top surface of the sample. For instance, Baskes et al. [46] proposed that permeation barriers made of, for example, Ti or Zr could be used to reduce the recombination coefficient by more than 10 orders of magnitude giving a significant increase in the total H concentration in the material. Another alternative could be to reduce the sample temperature. A higher temperature enhances diffusion and, thereby, reduces the maximum concentration [71].

Even though it is not possible to fully eliminate the gradient in the H concentration, the maximum solute concentration would be higher. Furthermore, the swift diffusion of H defines that only by using an in-situ charging approach any modification can be observed. Ex-situ charged samples would immediately return to their original condition, as shown in Figure 12.

## 5. Conclusions

The presented methodology allowed for having a direct observation of H effect on a CP steel during tensile tests, enabling monitoring the deformation and crack propagation with their correlation to the load-elongation curves. With more conventional charging methods, such as H gas or electrochemical charging, it can be very challenging to have in-situ charging and observation and, for such fast diffusing materials, only direct observation during in-situ charging, as established here, will allow addressing H effects on materials.

Different approaches were performed in order to investigate the behaviour of the steel under the effect of H. The combination of the applied charging conditions and a material exhibiting high H diffusivity resulted in a low H concentration, slightly below the critical value in order to produce embrittlement in the samples. Even though there was a constant supply of H, which led to a dynamic equilibrium of H concentration, this value was not sufficient to have a large impact on the mechanical properties of the material. When comparing these results with the H concentration absorbed during the body in white painting process, it can be concluded that the CP steel is a suitable material to build body in white components.

As aforementioned, there are already slight modifications in fracture characteristics and crack propagation rates, even noticeable at these concentrations. Furthermore, a number of possible future alternatives to generally increase the total H concentration in the material microstructure exist.

## Figures and Tables

**Table 1:** Chemical composition of the investigated industrial steel grade.

	C	Mn	Si	Cr	S	Nb	Ti	Al
wt%	<0.20	<2.6	<0.8	<1.00	<0.010	<0.05	<0.15	0.015–1.0

**Table 2:** Case studies and sample thicknesses.

Case Study	Overall Thickness (mm)	Center-Reduced Thickness (mm)
Rate Dependence of Flow Properties (Tensile Samples)	1.06 ± 0.01	–
Effect of H Pre-Charging Time	0.5 ± 0.01	0.12 ± 0.01
Effect of Plasma Parameters	1.10 ± 0.01	0.18 ± 0.01
Effect of H Charging-Discharging	1.10 ± 0.01	0.20 ± 0.01

**Table 3:** Recombination coefficient and lattice H concentration calculated with different sources.

Source	*k*_f_ (m^4^ s^−1^)	*C*_lattice_ (wppm)
Shu et al. [43]	1.75 × 10^−21^	1.31 × 10^−4^
Zhou et al. [45]	5.56 × 10^−21^	7.34 × 10^−5^
Baskes [46]	5.00 × 10^−24^	2.45 × 10^−3^
